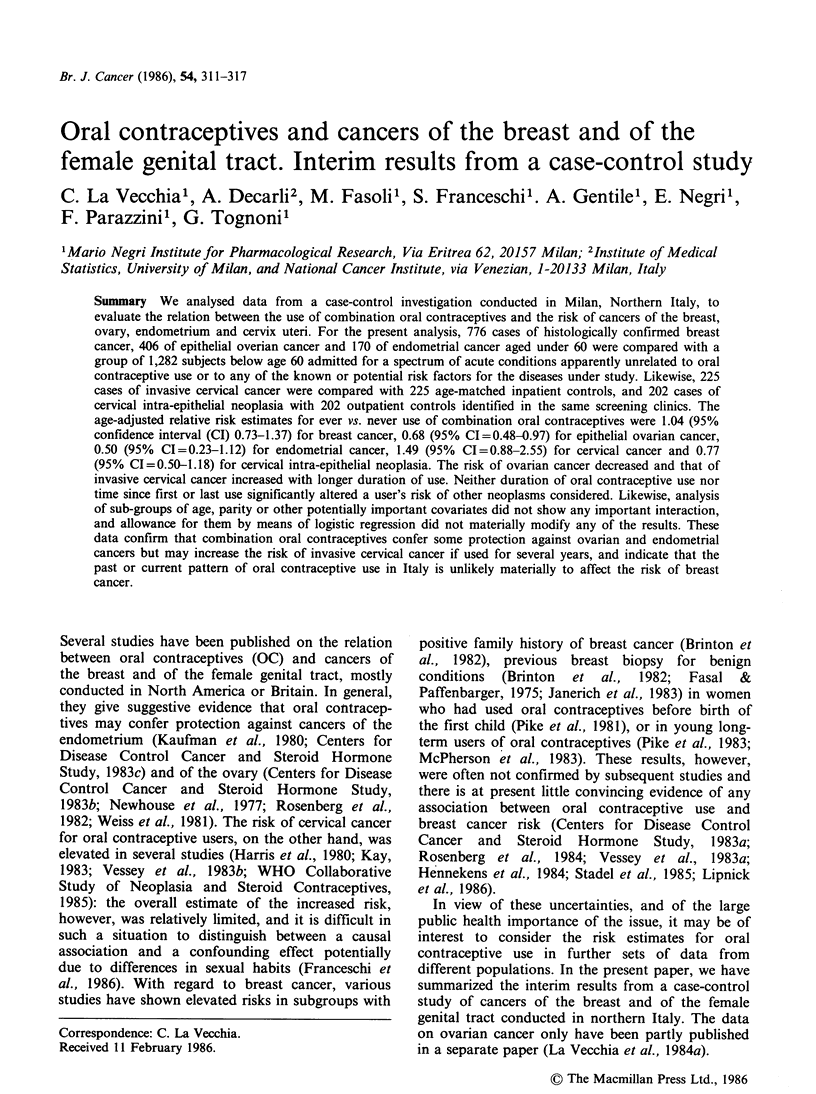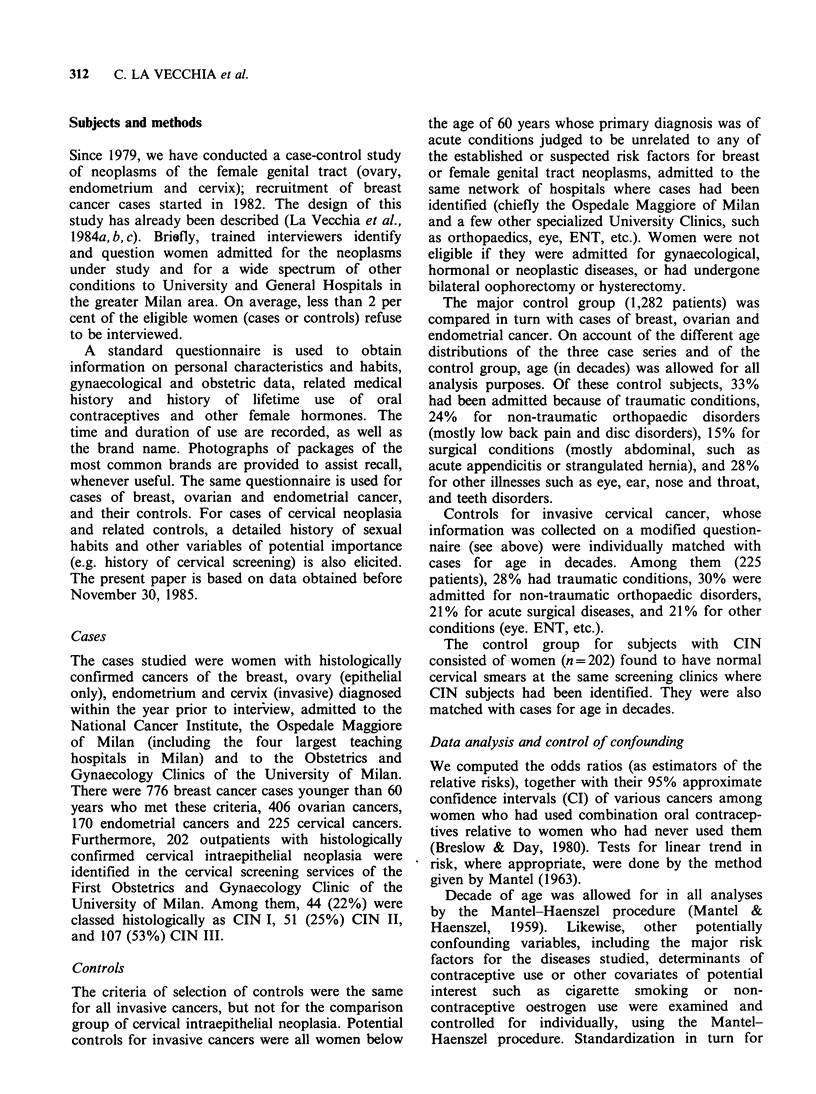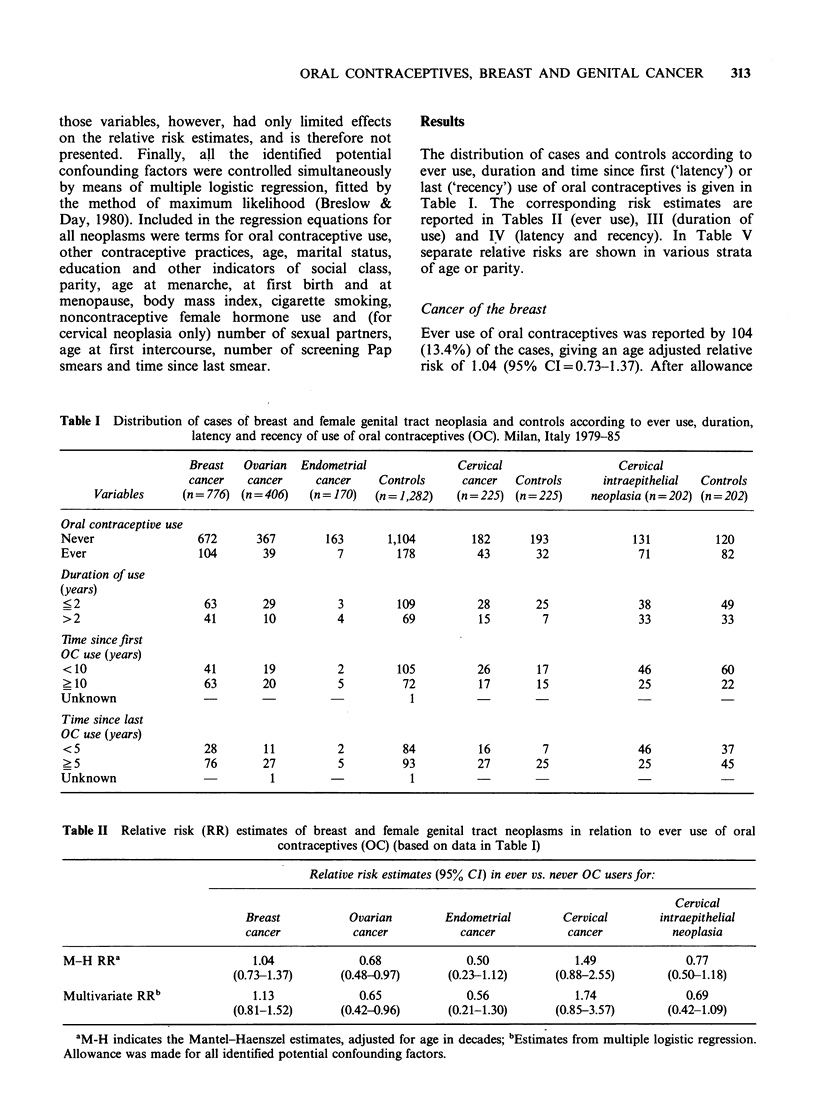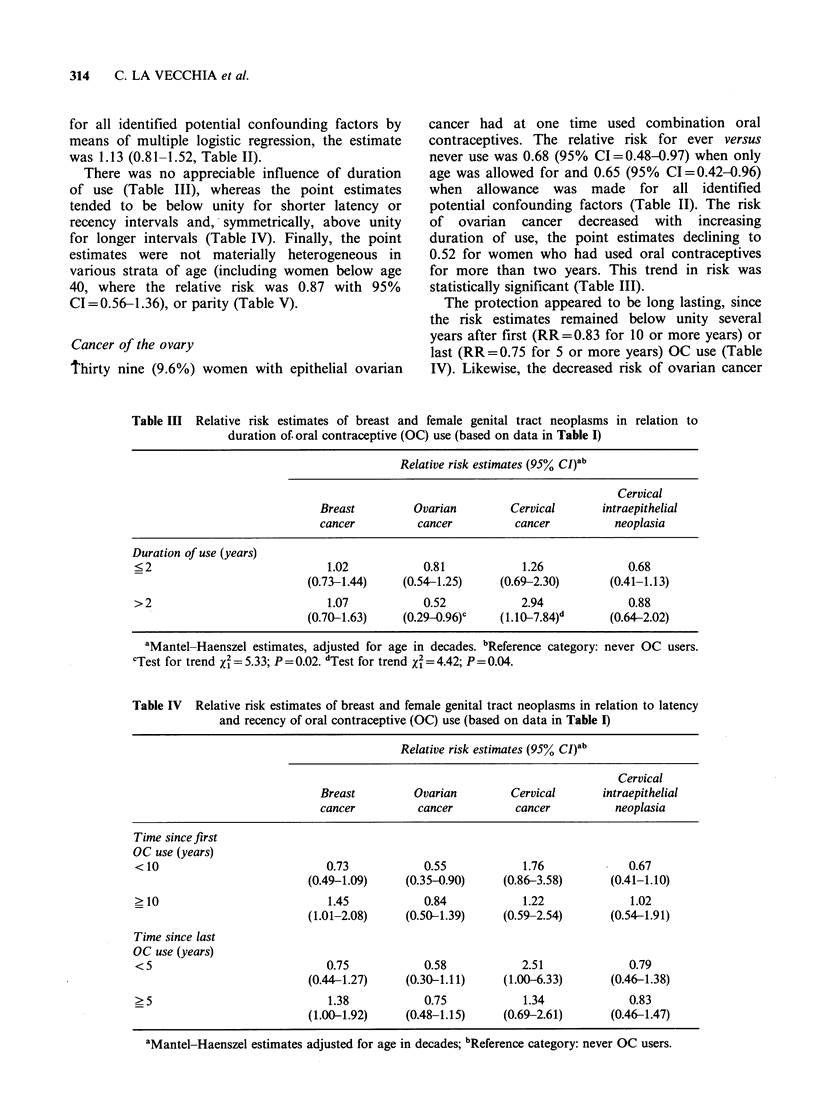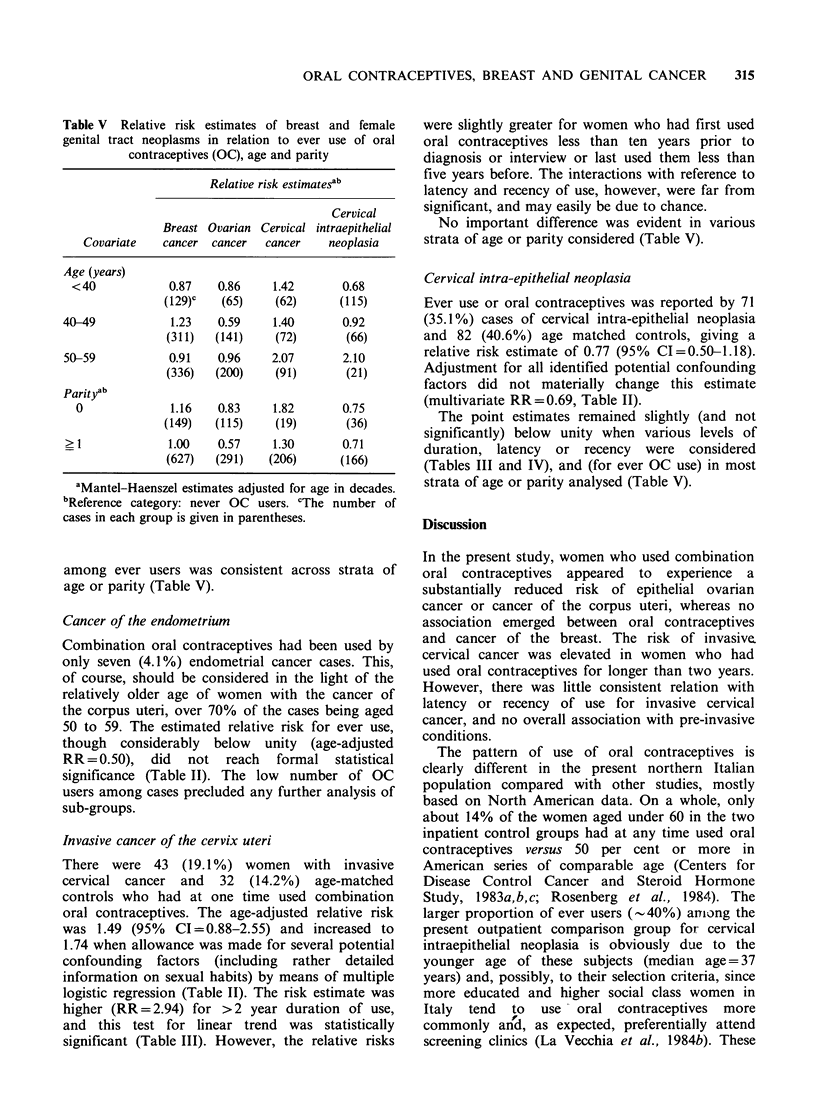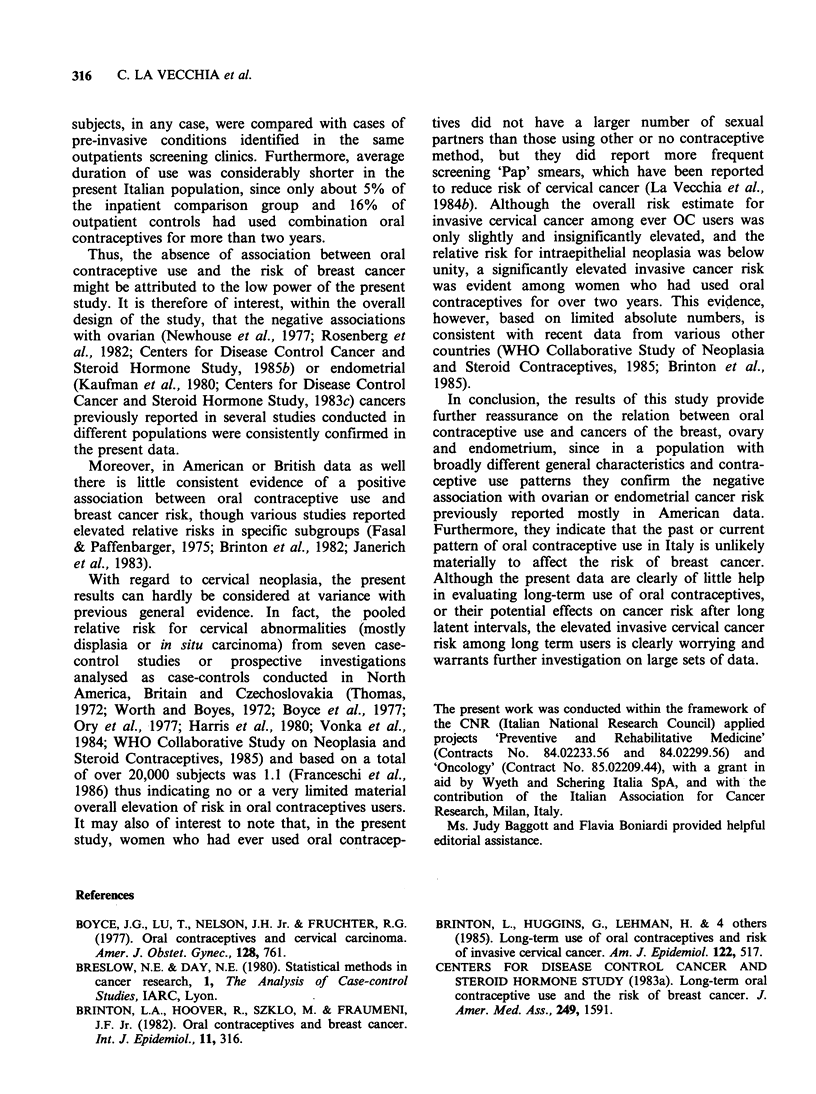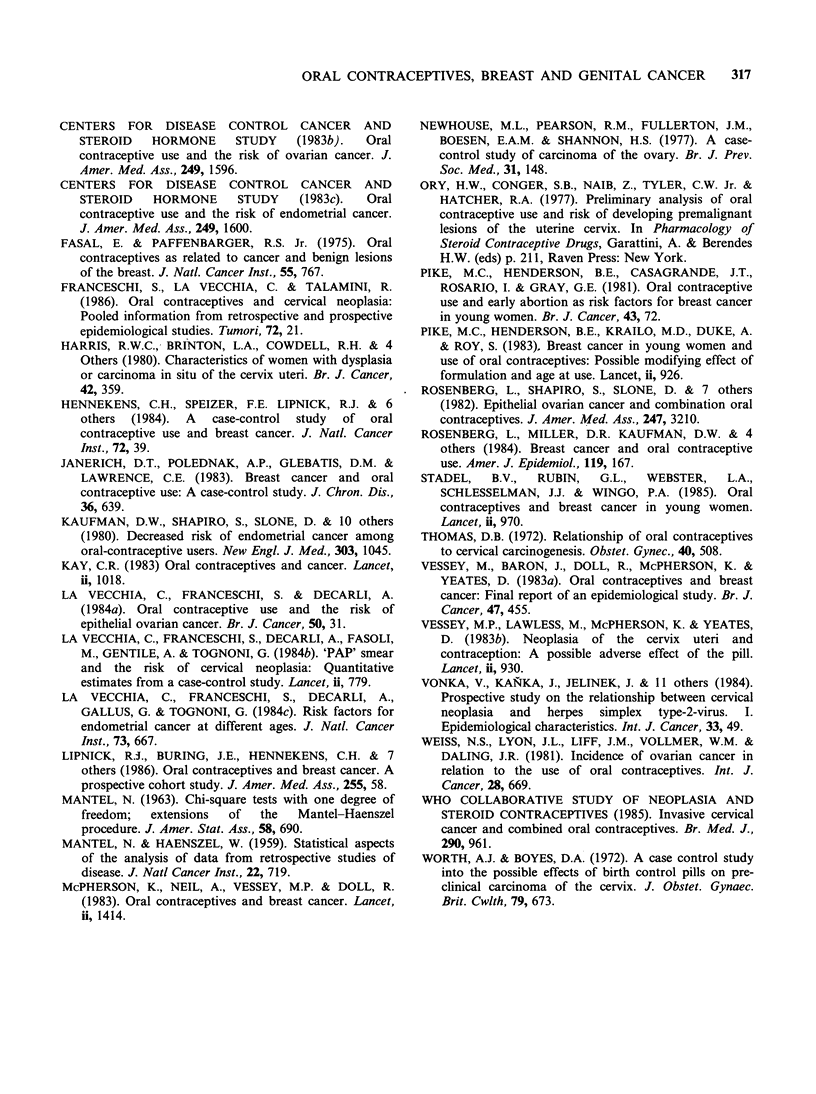# Oral contraceptives and cancers of the breast and of the female genital tract. Interim results from a case-control study.

**DOI:** 10.1038/bjc.1986.178

**Published:** 1986-08

**Authors:** C. La Vecchia, A. Decarli, M. Fasoli, S. Franceschi, A. Gentile, E. Negri, F. Parazzini, G. Tognoni

## Abstract

We analysed data from a case-control investigation conducted in Milan, Northern Italy, to evaluate the relation between the use of combination oral contraceptives and the risk of cancers of the breast, ovary, endometrium and cervix uteri. For the present analysis, 776 cases of histologically confirmed breast cancer, 406 of epithelial ovarian cancer and 170 of endometrial cancer aged under 60 were compared with a group of 1,282 subjects below age 60 admitted for a spectrum of acute conditions apparently unrelated to oral contraceptive use or to any of the known or potential risk factors for the diseases under study. Likewise, 225 cases of invasive cervical cancer were compared with 225 age-matched inpatient controls, and 202 cases of cervical intra-epithelial neoplasia with 202 outpatient controls identified in the same screening clinics. The age-adjusted relative risk estimates for ever vs. never use of combination oral contraceptives were 1.04 (95% confidence interval (CI) 0.73-1.37) for breast cancer, 0.68 (95% CI = 0.48-0.97) for epithelial ovarian cancer, 0.50 (95% CI = 0.23-1.12) for endometrial cancer, 1.49 (95% CI = 0.88-2.55) for cervical cancer and 0.77 (95% CI = 0.50-1.18) for cervical intra-epithelial neoplasia. The risk of ovarian cancer decreased and that of invasive cervical cancer increased with longer duration of use. Neither duration of oral contraceptive use nor time since first or last use significantly altered a user's risk of other neoplasms considered. Likewise, analysis of sub-groups of age, parity or other potentially important covariates did not show any important interaction, and allowance for them by means of logistic regression did not materially modify any of the results. These data confirm that combination oral contraceptives confer some protection against ovarian and endometrial cancers but may increase the risk of invasive cervical cancer if used for several years, and indicate that the past or current pattern of oral contraceptive use in Italy is unlikely materially to affect the risk of breast cancer.


*Br. J. Cancer* (1986), **54**, 311–317

### Oral contraceptives and cancers of the breast and of the female genital tract. Interim results from a case-control study

C. La Vecchia[1], A. Decarli[2], M. Fasoli[1], S. Franceschi[1]. A. Gentile[1], E. Negri[1], F. Parazzini[1], G. Tognoni[1]

[1]*Mario Negri Institute for Pharmacological Research, Via Eritrea 62, 20157 Milan;* [2]*Institute of Medical Statistics, University of Milan, and National Cancer Institute, via Venezian, 1-20133 Milan, Italy*

**Summary** We analysed data from a case-control investigation conducted in Milan, Northern Italy, to evaluate the relation between the use of combination oral contraceptives and the risk of cancers of the breast, ovary, endometrium and cervix uteri. For the present analysis, 776 cases of histologically confirmed breast cancer, 406 of epithelial overian cancer and 170 of endometrial cancer aged under 60 were compared with a group of 1,282 subjects below age 60 admitted for a spectrum of acute conditions apparently unrelated to oral contraceptive use or to any of the known or potential risk factors for the diseases under study. Likewise, 225 cases of invasive cervical cancer were compared with 225 age-matched inpatient controls, and 202 cases of cervical intra-epithelial neoplasia with 202 outpatient controls identified in the same screening clinics. The age-adjusted relative risk estimates for ever *vs.* never use of combination oral contraceptives were 1.04 (95% confidence interval (CI) 0.73–1.37) for breast cancer, 0.68 (95% CI = 0.48–0.97) for epithelial ovarian cancer, 0.50 (95% CI = 0.23–1.12) for endometrial cancer, 1.49 (95% CI = 0.88–2.55) for cervical cancer and 0.77 (95% CI = 0.50–1.18) for cervical intra-epithelial neoplasia. The risk of ovarian cancer decreased and that of invasive cervical cancer increased with longer duration of use. Neither duration of oral contraceptive use nor time since first or last use significantly altered a user's risk of other neoplasms considered. Likewise, analysis of sub-groups of age, parity or other potentially important covariates did not show any important interaction, and allowance for them by means of logistic regression did not materially modify any of the results. These data confirm that combination oral contraceptives confer some protection against ovarian and endometrial cancers but may increase the risk of invasive cervical cancer if used for several years, and indicate that the past or current pattern of oral contraceptive use in Italy is unlikely materially to affect the risk of breast cancer.

Several studies have been published on the relation between oral contraceptives (OC) and cancers of the breast and of the female genital tract, mostly conducted in North America or Britain. In general, they give suggestive evidence that oral contraceptives may confer protection against cancers of the endometrium (Kaufman *et al.*, 1980; Centers for Disease Control Cancer and Steroid Hormone Study, 1983*c*) and of the ovary (Centers for Disease Control Cancer and Steroid Hormone Study, 1983*b*; Newhouse *et al.*, 1977; Rosenberg *et al.*, 1982; Weiss *et al.*, 1981). The risk of cervical cancer for oral contraceptive users, on the other hand, was elevated in several studies (Harris *et al.*, 1980; Kay, 1983; Vessey *et al.*, 1983*b*; WHO Collaborative Study of Neoplasia and Steroid Contraceptives, 1985): the overall estimate of the increased risk, however, was relatively limited, and it is difficult in such a situation to distinguish between a causal association and a confounding effect potentially due to differences in sexual habits (Franceschi *et al.*, 1986). With regard to breast cancer, various studies have shown elevated risks in subgroups with positive family history of breast cancer (Brinton *et al.*, 1982), previous breast biopsy for benign conditions (Brinton *et al.*, 1982; Fasal & Paffenbarger, 1975; Janerich *et al.*, 1983) in women who had used oral contraceptives before birth of the first child (Pike *et al.*, 1981), or in young long-term users of oral contraceptives (Pike *et al.*, 1983; McPherson *et al.*, 1983). These results, however, were often not confirmed by subsequent studies and there is at present little convincing evidence of any association between oral contraceptive use and breast cancer risk (Centers for Disease Control Cancer and Steroid Hormone Study, 1983*a*; Rosenberg *et al.*, 1984; Vessey *et al.*, 1983*a*; Hennekens *et al.*, 1984; Stadel *et al.*, 1985; Lipnick *et al.*, 1986).

In view of these uncertainties, and of the large public health importance of the issue, it may be of interest to consider the risk estimates for oral contraceptive use in further sets of data from different populations. In the present paper, we have summarized the interim results from a case-control study of cancers of the breast and of the female genital tract conducted in northern Italy. The data on ovarian cancer only have been partly published in a separate paper (La Vecchia *et al.*, 1984*a*).

Correspondence: C. La Vecchia.
Received 11 February 1986.

© The Macmillan Press Ltd., 1986

### Subjects and methods

Since 1979, we have conducted a case-control study of neoplasms of the female genital tract (ovary, endometrium and cervix); recruitment of breast cancer cases started in 1982. The design of this study has already been described (La Vecchia *et al.*, 1984*a, b, c*). Briefly, trained interviewers identify and question women admitted for the neoplasms under study and for a wide spectrum of other conditions to University and General Hospitals in the greater Milan area. On average, less than 2 per cent of the eligible women (cases or controls) refuse to be interviewed.

A standard questionnaire is used to obtain information on personal characteristics and habits, gynaecological and obstetric data, related medical history and history of lifetime use of oral contraceptives and other female hormones. The time and duration of use are recorded, as well as the brand name. Photographs of packages of the most common brands are provided to assist recall, whenever useful. The same questionnaire is used for cases of breast, ovarian and endometrial cancer, and their controls. For cases of cervical neoplasia and related controls, a detailed history of sexual habits and other variables of potential importance (e.g. history of cervical screening) is also elicited. The present paper is based on data obtained before November 30, 1985.

### *Cases*

The cases studied were women with histologically confirmed cancers of the breast, ovary (epithelial only), endometrium and cervix (invasive) diagnosed within the year prior to interview, admitted to the National Cancer Institute, the Ospedale Maggiore of Milan (including the four largest teaching hospitals in Milan) and to the Obstetrics and Gynaecology Clinics of the University of Milan. There were 776 breast cancer cases younger than 60 years who met these criteria, 406 ovarian cancers, 170 endometrial cancers and 225 cervical cancers. Furthermore, 202 outpatients with histologically confirmed cervical intraepithelial neoplasia were identified in the cervical screening services of the First Obstetrics and Gynaecology Clinic of the University of Milan. Among them, 44 (22%) were classed histologically as CIN I, 51 (25%) CIN II, and 107 (53%) CIN III.

### *Controls*

The criteria of selection of controls were the same for all invasive cancers, but not for the comparison group of cervical intraepithelial neoplasia. Potential controls for invasive cancers were all women below the age of 60 years whose primary diagnosis was of acute conditions judged to be unrelated to any of the established or suspected risk factors for breast or female genital tract neoplasms, admitted to the same network of hospitals where cases had been identified (chiefly the Ospedale Maggiore of Milan and a few other specialized University Clinics, such as orthopaedics, eye, ENT, etc.). Women were not eligible if they were admitted for gynaecological, hormonal or neoplastic diseases, or had undergone bilateral oophorectomy or hysterectomy.

The major control group (1,282 patients) was compared in turn with cases of breast, ovarian and endometrial cancer. On account of the different age distributions of the three case series and of the control group, age (in decades) was allowed for all analysis purposes. Of these control subjects, 33% had been admitted because of traumatic conditions, 24% for non-traumatic orthopaedic disorders (mostly low back pain and disc disorders), 15% for surgical conditions (mostly abdominal, such as acute appendicitis or strangulated hernia), and 28% for other illnesses such as eye, ear, nose and throat, and teeth disorders.

Controls for invasive cervical cancer, whose information was collected on a modified questionnaire (see above) were individually matched with cases for age in decades. Among them (225 patients), 28% had traumatic conditions, 30% were admitted for non-traumatic orthopaedic disorders, 21% for acute surgical diseases, and 21% for other conditions (eye. ENT, etc.).

The control group for subjects with CIN consisted of women ($n=202$) found to have normal cervical smears at the same screening clinics where CIN subjects had been identified. They were also matched with cases for age in decades.

### *Data analysis and control of confounding*

We computed the odds ratios (as estimators of the relative risks), together with their 95% approximate confidence intervals (CI) of various cancers among women who had used combination oral contraceptives relative to women who had never used them (Breslow & Day, 1980). Tests for linear trend in risk, where appropriate, were done by the method given by Mantel (1963).

Decade of age was allowed for in all analyses by the Mantel–Haenszel procedure (Mantel & Haenszel, 1959). Likewise, other potentially confounding variables, including the major risk factors for the diseases studied, determinants of contraceptive use or other covariates of potential interest such as cigarette smoking or non-contraceptive oestrogen use were examined and controlled for individually, using the Mantel–Haenszel procedure. Standardization in turn for those variables, however, had only limited effects on the relative risk estimates, and is therefore not presented. Finally, all the identified potential confounding factors were controlled simultaneously by means of multiple logistic regression, fitted by the method of maximum likelihood (Breslow & Day, 1980). Included in the regression equations for all neoplasms were terms for oral contraceptive use, other contraceptive practices, age, marital status, education and other indicators of social class, parity, age at menarche, at first birth and at menopause, body mass index, cigarette smoking, noncontraceptive female hormone use and (for cervical neoplasia only) number of sexual partners, age at first intercourse, number of screening Pap smears and time since last smear.

### Results

The distribution of cases and controls according to ever use, duration and time since first ('latency') or last ('recency') use of oral contraceptives is given in Table I. The corresponding risk estimates are reported in Tables II (ever use), III (duration of use) and IV (latency and recency). In Table V separate relative risks are shown in various strata of age or parity.

### *Cancer of the breast*

Ever use of oral contraceptives was reported by 104 (13.4%) of the cases, giving an age adjusted relative risk of 1.04 (95% CI=0.73–1.37). After allowance

**Table I** Distribution of cases of breast and female genital tract neoplasia and controls according to ever use, duration, latency and recency of use of oral contraceptives (OC). Milan, Italy 1979–85

| *Variables* | *Breast cancer (n=776)* | *Ovarian cancer (n=406)* | *Endometrial cancer (n=170)* | *Controls (n=1,282)* | *Cervical cancer (n=225)* | *Controls (n=225)* | *Cervical intraepithelial neoplasia (n=202)* | *Controls (n=202)* |
|---|---|---|---|---|---|---|---|---|
| *Oral contraceptive use* | | | | | | | | |
| Never | 672 | 367 | 163 | 1,104 | 182 | 193 | 131 | 120 |
| Ever | 104 | 39 | 7 | 178 | 43 | 32 | 71 | 82 |
| *Duration of use (years)* | | | | | | | | |
| ≦2 | 63 | 29 | 3 | 109 | 28 | 25 | 38 | 49 |
| >2 | 41 | 10 | 4 | 69 | 15 | 7 | 33 | 33 |
| *Time since first OC use (years)* | | | | | | | | |
| <10 | 41 | 19 | 2 | 105 | 26 | 17 | 46 | 60 |
| ≧10 | 63 | 20 | 5 | 72 | 17 | 15 | 25 | 22 |
| Unknown | — | — | — | 1 | — | — | — | — |
| *Time since last OC use (years)* | | | | | | | | |
| <5 | 28 | 11 | 2 | 84 | 16 | 7 | 46 | 37 |
| ≧5 | 76 | 27 | 5 | 93 | 27 | 25 | 25 | 45 |
| Unknown | — | 1 | — | 1 | — | — | — | — |

**Table II** Relative risk (RR) estimates of breast and female genital tract neoplasms in relation to ever use of oral contraceptives (OC) (based on data in Table I)

| | *Relative risk estimates (95% CI) in ever vs. never OC users for:* | | | | |
|---|---|---|---|---|---|
| | *Breast cancer* | *Ovarian cancer* | *Endometrial cancer* | *Cervical cancer* | *Cervical intraepithelial neoplasia* |
| M-H RR[a] | 1.04 (0.73–1.37) | 0.68 (0.48–0.97) | 0.50 (0.23–1.12) | 1.49 (0.88–2.55) | 0.77 (0.50–1.18) |
| Multivariate RR[b] | 1.13 (0.81–1.52) | 0.65 (0.42–0.96) | 0.56 (0.21–1.30) | 1.74 (0.85–3.57) | 0.69 (0.42–1.09) |

[a]M-H indicates the Mantel–Haenszel estimates, adjusted for age in decades; [b]Estimates from multiple logistic regression. Allowance was made for all identified potential confounding factors.

for all identified potential confounding factors by means of multiple logistic regression, the estimate was 1.13 (0.81–1.52, Table II).

There was no appreciable influence of duration of use (Table III), whereas the point estimates tended to be below unity for shorter latency or recency intervals and, symmetrically, above unity for longer intervals (Table IV). Finally, the point estimates were not materially heterogeneous in various strata of age (including women below age 40, where the relative risk was 0.87 with 95% CI = 0.56–1.36), or parity (Table V).

*Cancer of the ovary*

Thirty nine (9.6%) women with epithelial ovarian cancer had at one time used combination oral contraceptives. The relative risk for ever *versus* never use was 0.68 (95% CI = 0.48–0.97) when only age was allowed for and 0.65 (95% CI = 0.42–0.96) when allowance was made for all identified potential confounding factors (Table II). The risk of ovarian cancer decreased with increasing duration of use, the point estimates declining to 0.52 for women who had used oral contraceptives for more than two years. This trend in risk was statistically significant (Table III).

The protection appeared to be long lasting, since the risk estimates remained below unity several years after first (RR = 0.83 for 10 or more years) or last (RR = 0.75 for 5 or more years) OC use (Table IV). Likewise, the decreased risk of ovarian cancer

**Table III** Relative risk estimates of breast and female genital tract neoplasms in relation to duration of oral contraceptive (OC) use (based on data in **Table I**)

| | *Relative risk estimates (95% CI)*[a,b] | | | |
|---|---|---|---|---|
| | *Breast cancer* | *Ovarian cancer* | *Cervical cancer* | *Cervical intraepithelial neoplasia* |
| *Duration of use (years)* | | | | |
| ≦2 | 1.02<br>(0.73–1.44) | 0.81<br>(0.54–1.25) | 1.26<br>(0.69–2.30) | 0.68<br>(0.41–1.13) |
| >2 | 1.07<br>(0.70–1.63) | 0.52<br>(0.29–0.96)[c] | 2.94<br>(1.10–7.84)[d] | 0.88<br>(0.64–2.02) |

[a]Mantel–Haenszel estimates, adjusted for age in decades. [b]Reference category: never OC users. [c]Test for trend $\chi_1^2 = 5.33$; $P = 0.02$. [d]Test for trend $\chi_1^2 = 4.42$; $P = 0.04$.

**Table IV** Relative risk estimates of breast and female genital tract neoplasms in relation to latency and recency of oral contraceptive (OC) use (based on data in **Table I**)

| | *Relative risk estimates (95% CI)*[a,b] | | | |
|---|---|---|---|---|
| | *Breast cancer* | *Ovarian cancer* | *Cervical cancer* | *Cervical intraepithelial neoplasia* |
| *Time since first OC use (years)* | | | | |
| <10 | 0.73<br>(0.49–1.09) | 0.55<br>(0.35–0.90) | 1.76<br>(0.86–3.58) | 0.67<br>(0.41–1.10) |
| ≧10 | 1.45<br>(1.01–2.08) | 0.84<br>(0.50–1.39) | 1.22<br>(0.59–2.54) | 1.02<br>(0.54–1.91) |
| *Time since last OC use (years)* | | | | |
| <5 | 0.75<br>(0.44–1.27) | 0.58<br>(0.30–1.11) | 2.51<br>(1.00–6.33) | 0.79<br>(0.46–1.38) |
| ≧5 | 1.38<br>(1.00–1.92) | 0.75<br>(0.48–1.15) | 1.34<br>(0.69–2.61) | 0.83<br>(0.46–1.47) |

[a]Mantel–Haenszel estimates adjusted for age in decades; [b]Reference category: never OC users.

**Table V** Relative risk estimates of breast and female genital tract neoplasms in relation to ever use of oral contraceptives (OC), age and parity

| | *Relative risk estimates*[ab] | | | |
|---|---|---|---|---|
| *Covariate* | *Breast cancer* | *Ovarian cancer* | *Cervical cancer* | *Cervical intraepithelial neoplasia* |
| *Age (years)* | | | | |
| <40 | 0.87 (129)[c] | 0.86 (65) | 1.42 (62) | 0.68 (115) |
| 40–49 | 1.23 (311) | 0.59 (141) | 1.40 (72) | 0.92 (66) |
| 50–59 | 0.91 (336) | 0.96 (200) | 2.07 (91) | 2.10 (21) |
| *Parity*[ab] | | | | |
| 0 | 1.16 (149) | 0.83 (115) | 1.82 (19) | 0.75 (36) |
| ≥1 | 1.00 (627) | 0.57 (291) | 1.30 (206) | 0.71 (166) |

[a]Mantel–Haenszel estimates adjusted for age in decades. [b]Reference category: never OC users. [c]The number of cases in each group is given in parentheses.

among ever users was consistent across strata of age or parity (Table V).

*Cancer of the endometrium*

Combination oral contraceptives had been used by only seven (4.1%) endometrial cancer cases. This, of course, should be considered in the light of the relatively older age of women with the cancer of the corpus uteri, over 70% of the cases being aged 50 to 59. The estimated relative risk for ever use, though considerably below unity (age-adjusted RR = 0.50), did not reach formal statistical significance (Table II). The low number of OC users among cases precluded any further analysis of sub-groups.

*Invasive cancer of the cervix uteri*

There were 43 (19.1%) women with invasive cervical cancer and 32 (14.2%) age-matched controls who had at one time used combination oral contraceptives. The age-adjusted relative risk was 1.49 (95% CI = 0.88–2.55) and increased to 1.74 when allowance was made for several potential confounding factors (including rather detailed information on sexual habits) by means of multiple logistic regression (Table II). The risk estimate was higher (RR = 2.94) for >2 year duration of use, and this test for linear trend was statistically significant (Table III). However, the relative risks were slightly greater for women who had first used oral contraceptives less than ten years prior to diagnosis or interview or last used them less than five years before. The interactions with reference to latency and recency of use, however, were far from significant, and may easily be due to chance.

No important difference was evident in various strata of age or parity considered (Table V).

*Cervical intra-epithelial neoplasia*

Ever use or oral contraceptives was reported by 71 (35.1%) cases of cervical intra-epithelial neoplasia and 82 (40.6%) age matched controls, giving a relative risk estimate of 0.77 (95% CI = 0.50–1.18). Adjustment for all identified potential confounding factors did not materially change this estimate (multivariate RR = 0.69, Table II).

The point estimates remained slightly (and not significantly) below unity when various levels of duration, latency or recency were considered (Tables III and IV), and (for ever OC use) in most strata of age or parity analysed (Table V).

### Discussion

In the present study, women who used combination oral contraceptives appeared to experience a substantially reduced risk of epithelial ovarian cancer or cancer of the corpus uteri, whereas no association emerged between oral contraceptives and cancer of the breast. The risk of invasive cervical cancer was elevated in women who had used oral contraceptives for longer than two years. However, there was little consistent relation with latency or recency of use for invasive cervical cancer, and no overall association with pre-invasive conditions.

The pattern of use of oral contraceptives is clearly different in the present northern Italian population compared with other studies, mostly based on North American data. On a whole, only about 14% of the women aged under 60 in the two inpatient control groups had at any time used oral contraceptives *versus* 50 per cent or more in American series of comparable age (Centers for Disease Control Cancer and Steroid Hormone Study, 1983*a,b,c*; Rosenberg *et al.*, 1984). The larger proportion of ever users (~40%) among the present outpatient comparison group for cervical intraepithelial neoplasia is obviously due to the younger age of these subjects (median age = 37 years) and, possibly, to their selection criteria, since more educated and higher social class women in Italy tend to use oral contraceptives more commonly and, as expected, preferentially attend screening clinics (La Vecchia *et al.*, 1984*b*). These subjects, in any case, were compared with cases of pre-invasive conditions identified in the same outpatients screening clinics. Furthermore, average duration of use was considerably shorter in the present Italian population, since only about 5% of the inpatient comparison group and 16% of outpatient controls had used combination oral contraceptives for more than two years.

Thus, the absence of association between oral contraceptive use and the risk of breast cancer might be attributed to the low power of the present study. It is therefore of interest, within the overall design of the study, that the negative associations with ovarian (Newhouse *et al.*, 1977; Rosenberg *et al.*, 1982; Centers for Disease Control Cancer and Steroid Hormone Study, 1985*b*) or endometrial (Kaufman *et al.*, 1980; Centers for Disease Control Cancer and Steroid Hormone Study, 1983*c*) cancers previously reported in several studies conducted in different populations were consistently confirmed in the present data.

Moreover, in American or British data as well there is little consistent evidence of a positive association between oral contraceptive use and breast cancer risk, though various studies reported elevated relative risks in specific subgroups (Fasal & Paffenbarger, 1975; Brinton *et al.*, 1982; Janerich *et al.*, 1983).

With regard to cervical neoplasia, the present results can hardly be considered at variance with previous general evidence. In fact, the pooled relative risk for cervical abnormalities (mostly displasia or *in situ* carcinoma) from seven case-control studies or prospective investigations analysed as case-controls conducted in North America, Britain and Czechoslovakia (Thomas, 1972; Worth and Boyes, 1972; Boyce *et al.*, 1977; Ory *et al.*, 1977; Harris *et al.*, 1980; Vonka *et al.*, 1984; WHO Collaborative Study on Neoplasia and Steroid Contraceptives, 1985) and based on a total of over 20,000 subjects was 1.1 (Franceschi *et al.*, 1986) thus indicating no or a very limited material overall elevation of risk in oral contraceptives users. It may also of interest to note that, in the present study, women who had ever used oral contraceptives did not have a larger number of sexual partners than those using other or no contraceptive method, but they did report more frequent screening 'Pap' smears, which have been reported to reduce risk of cervical cancer (La Vecchia *et al.*, 1984*b*). Although the overall risk estimate for invasive cervical cancer among ever OC users was only slightly and insignificantly elevated, and the relative risk for intraepithelial neoplasia was below unity, a significantly elevated invasive cancer risk was evident among women who had used oral contraceptives for over two years. This evidence, however, based on limited absolute numbers, is consistent with recent data from various other countries (WHO Collaborative Study of Neoplasia and Steroid Contraceptives, 1985; Brinton *et al.*, 1985).

In conclusion, the results of this study provide further reassurance on the relation between oral contraceptive use and cancers of the breast, ovary and endometrium, since in a population with broadly different general characteristics and contraceptive use patterns they confirm the negative association with ovarian or endometrial cancer risk previously reported mostly in American data. Furthermore, they indicate that the past or current pattern of oral contraceptive use in Italy is unlikely materially to affect the risk of breast cancer. Although the present data are clearly of little help in evaluating long-term use of oral contraceptives, or their potential effects on cancer risk after long latent intervals, the elevated invasive cervical cancer risk among long term users is clearly worrying and warrants further investigation on large sets of data.

The present work was conducted within the framework of the CNR (Italian National Research Council) applied projects 'Preventive and Rehabilitative Medicine' (Contracts No. 84.02233.56 and 84.02299.56) and 'Oncology' (Contract No. 85.02209.44), with a grant in aid by Wyeth and Schering Italia SpA, and with the contribution of the Italian Association for Cancer Research, Milan, Italy.

Ms. Judy Baggott and Flavia Boniardi provided helpful editorial assistance.

### References

BOYCE, J.G., LU, T., NELSON, J.H. Jr. & FRUCHTER, R.G. (1977). Oral contraceptives and cervical carcinoma. *Amer. J. Obstet. Gynec.*, **128,** 761.

BRESLOW, N.E. & DAY, N.E. (1980). Statistical methods in cancer research, **1,** *The Analysis of Case-control Studies*, IARC, Lyon.

BRINTON, L.A., HOOVER, R., SZKLO, M. & FRAUMENI, J.F. Jr. (1982). Oral contraceptives and breast cancer. *Int. J. Epidemiol.*, **11,** 316.

BRINTON, L., HUGGINS, G., LEHMAN, H. & 4 others (1985). Long-term use of oral contraceptives and risk of invasive cervical cancer. *Am. J. Epidemiol.* **122,** 517.

CENTERS FOR DISEASE CONTROL CANCER AND STEROID HORMONE STUDY (1983a). Long-term oral contraceptive use and the risk of breast cancer. *J. Amer. Med. Ass.*, **249,** 1591.

CENTERS FOR DISEASE CONTROL CANCER AND STEROID HORMONE STUDY (1983*b*). Oral contraceptive use and the risk of ovarian cancer. *J. Amer. Med. Ass.*, **249**, 1596.

CENTERS FOR DISEASE CONTROL CANCER AND STEROID HORMONE STUDY (1983*c*). Oral contraceptive use and the risk of endometrial cancer. *J. Amer. Med. Ass.*, **249**, 1600.

FASAL, E. & PAFFENBARGER, R.S. Jr. (1975). Oral contraceptives as related to cancer and benign lesions of the breast. *J. Natl. Cancer Inst.*, **55**, 767.

FRANCESCHI, S., LA VECCHIA, C. & TALAMINI, R. (1986). Oral contraceptives and cervical neoplasia: Pooled information from retrospective and prospective epidemiological studies. *Tumori*, **72**, 21.

HARRIS, R.W.C., BRINTON, L.A., COWDELL, R.H. & 4 Others (1980). Characteristics of women with dysplasia or carcinoma in situ of the cervix uteri. *Br. J. Cancer*, **42**, 359.

HENNEKENS, C.H., SPEIZER, F.E. LIPNICK, R.J. & 6 others (1984). A case-control study of oral contraceptive use and breast cancer. *J. Natl. Cancer Inst.*, **72**, 39.

JANERICH, D.T., POLEDNAK, A.P., GLEBATIS, D.M. & LAWRENCE, C.E. (1983). Breast cancer and oral contraceptive use: A case-control study. *J. Chron. Dis.*, **36**, 639.

KAUFMAN, D.W., SHAPIRO, S., SLONE, D. & 10 others (1980). Decreased risk of endometrial cancer among oral-contraceptive users. *New Engl. J. Med.*, **303**, 1045.

KAY, C.R. (1983) Oral contraceptives and cancer. *Lancet*, **ii**, 1018.

LA VECCHIA, C., FRANCESCHI, S. & DECARLI, A. (1984*a*). Oral contraceptive use and the risk of epithelial ovarian cancer. *Br. J. Cancer*, **50**, 31.

LA VECCHIA, C., FRANCESCHI, S., DECARLI, A., FASOLI, M., GENTILE, A. & TOGNONI, G. (1984*b*). 'PAP' smear and the risk of cervical neoplasia: Quantitative estimates from a case-control study. *Lancet*, **ii**, 779.

LA VECCHIA, C., FRANCESCHI, S., DECARLI, A., GALLUS, G. & TOGNONI, G. (1984*c*). Risk factors for endometrial cancer at different ages. *J. Natl. Cancer Inst.*, **73**, 667.

LIPNICK, R.J., BURING, J.E., HENNEKENS, C.H. & 7 others (1986). Oral contraceptives and breast cancer. A prospective cohort study. *J. Amer. Med. Ass.*, **255**, 58.

MANTEL, N. (1963). Chi-square tests with one degree of freedom; extensions of the Mantel–Haenszel procedure. *J. Amer. Stat. Ass.*, **58**, 690.

MANTEL, N. & HAENSZEL, W. (1959). Statistical aspects of the analysis of data from retrospective studies of disease. *J. Natl Cancer Inst.*, **22**, 719.

McPHERSON, K., NEIL, A., VESSEY, M.P. & DOLL, R. (1983). Oral contraceptives and breast cancer. *Lancet*, **ii**, 1414.

NEWHOUSE, M.L., PEARSON, R.M., FULLERTON, J.M., BOESEN, E.A.M. & SHANNON, H.S. (1977). A case-control study of carcinoma of the ovary. *Br. J. Prev. Soc. Med.*, **31**, 148.

ORY, H.W., CONGER, S.B., NAIB, Z., TYLER, C.W. Jr. & HATCHER, R.A. (1977). Preliminary analysis of oral contraceptive use and risk of developing premalignant lesions of the uterine cervix. In *Pharmacology of Steroid Contraceptive Drugs*, Garattini, A. & Berendes H.W. (eds) p. 211, Raven Press: New York.

PIKE, M.C., HENDERSON, B.E., CASAGRANDE, J.T., ROSARIO, I. & GRAY, G.E. (1981). Oral contraceptive use and early abortion as risk factors for breast cancer in young women. *Br. J. Cancer*, **43**, 72.

PIKE, M.C., HENDERSON, B.E., KRAILO, M.D., DUKE, A. & ROY, S. (1983). Breast cancer in young women and use of oral contraceptives: Possible modifying effect of formulation and age at use. Lancet, **ii**, 926.

ROSENBERG, L., SHAPIRO, S., SLONE, D. & 7 others (1982). Epithelial ovarian cancer and combination oral contraceptives. *J. Amer. Med. Ass.*, **247**, 3210.

ROSENBERG, L., MILLER, D.R. KAUFMAN, D.W. & 4 others (1984). Breast cancer and oral contraceptive use. *Amer. J. Epidemiol.*, **119**, 167.

STADEL, B.V., RUBIN, G.L., WEBSTER, L.A., SCHLESSELMAN, J.J. & WINGO, P.A. (1985). Oral contraceptives and breast cancer in young women. *Lancet*, **ii**, 970.

THOMAS, D.B. (1972). Relationship of oral contraceptives to cervical carcinogenesis. *Obstet. Gynec.*, **40**, 508.

VESSEY, M., BARON, J., DOLL, R., McPHERSON, K. & YEATES, D. (1983*a*). Oral contraceptives and breast cancer: Final report of an epidemiological study. *Br. J. Cancer*, **47**, 455.

VESSEY, M.P., LAWLESS, M., McPHERSON, K. & YEATES, D. (1983*b*). Neoplasia of the cervix uteri and contraception: A possible adverse effect of the pill. *Lancet*, **ii**, 930.

VONKA, V., KAŇKA, J., JELINEK, J. & 11 others (1984). Prospective study on the relationship between cervical neoplasia and herpes simplex type-2-virus. I. Epidemiological characteristics. *Int. J. Cancer*, **33**, 49.

WEISS, N.S., LYON, J.L., LIFF, J.M., VOLLMER, W.M. & DALING, J.R. (1981). Incidence of ovarian cancer in relation to the use of oral contraceptives. *Int. J. Cancer*, **28**, 669.

WHO COLLABORATIVE STUDY OF NEOPLASIA AND STEROID CONTRACEPTIVES (1985). Invasive cervical cancer and combined oral contraceptives. *Br. Med. J.*, **290**, 961.

WORTH, A.J. & BOYES, D.A. (1972). A case control study into the possible effects of birth control pills on pre-clinical carcinoma of the cervix. *J. Obstet. Gynaec. Brit. Cwlth*, **79**, 673.